# c-Myc alters the DNA damage-induced G2/M arrest in human mammary epithelial cells

**DOI:** 10.1038/sj.bjc.6601307

**Published:** 2003-10-14

**Authors:** J-H Sheen, J-K Woo, R B Dickson

**Affiliations:** 1 Department of Oncology, Lombardi Comprehensive Cancer Center, Room W417B, Georgetown University Medical Center, 3970 Reservoir Road, Washington DC 20007-2197, USA

**Keywords:** c-Myc, DNA damage, ionising radiation, G2/M checkpoint, cyclin B1, human mammary epithelial cells

## Abstract

Effects of c-Myc overexpression on the DNA damage-induced G2/M checkpoint were studied in finite lifespan, normal human mammary epithelial cells (HMECs). Previously, we showed that c-Myc attenuates G1/S arrest and leads to an inappropriate entry of cells with damaged DNA into the S phase, following treatment with ionising radiation (IR). Here we show that, in striking contrast to control cells, c-Myc-overexpressing HMECs demonstrate a significant attenuation of the G2/M arrest, following IR, and enter into inappropriate mitoses. At the molecular level, ectopic overexpression of c-Myc leads to an unusually high level of expression of cyclin B1, and the elevated levels of cyclin B1 were maintained, after *γ*-irradiation. Introduction of DNA damage in c-Myc-overexpressing, normal mammary epithelial cells eventually induces apoptosis, indicating a dramatic sensitisation by c-Myc of DNA damage-induced apoptosis. These two remarkable phenotypes, checkpoint attenuation and sensitisation to apoptosis, resulting from a deregulation of the protooncogene c-*myc*, may produce a unique pattern of alternating cycles, consisting first of amplification of DNA damage, followed by apoptosis-assisted selective pressure. The result of this alternating pattern of damage apoptosis could facilitate the selection of certain genomic alterations required for cellular survival and cellular transformation.

In response to DNA damage, cells arrest primarily at the G1/S and G2/M boundaries (Hartwell *et al*, 1994; Kastan, 2001). G1/S arrest blocks entry of damaged DNA into the S phase, and G2/M arrest prohibits entry of damaged DNA into mitosis. Therefore, both G1/S and G2/M checkpoints are important for maintaining genomic stability, an essential requirement for the fidelity of transmission of intact genomes to cellular progeny (Hartwell, 1992; Paulovich *et al*, 1997). The G2/M checkpoint appears to be established through two distinct steps: immediate establishment of the checkpoint, and maintenance of prolonged arrest (Taylor and Stark, 2001). The eventual target of the G2/M-entry checkpoint is the mitotic kinase, Cdc2-cyclin B, which governs most of the processes involved in mitotic initiation and progression (Murray and Hunt, 1993). In response to DNA damage, cells prevent the activation of Cdc2-cyclin B through various mechanisms, including cytoplasmic sequestration of critical components for Cdc2 activation (cyclin B and Cdc25C), transactivation of the cyclin-dependent kinase inhibitor (CKI) p21/Cip1, and transrepression of Cdc2 and cyclin B (Bunz *et al*, 1998; Taylor *et al*, 1999; Graves *et al*, 2001). For initial establishment of the G2/M checkpoint, elimination of the Cdc25C protein phosphatase activity appears to be a critical step (Peng *et al*, 1997; Sanchez *et al*, 1997; Zeng and Piwnica-Worms, 1999; Graves *et al*, 2001; Zhao and Piwnica-Worms, 2001). A well-known tumour suppressor, p53 protein, has also been demonstrated to participate in the maintenance of the G2/M checkpoint. p53-defective cells fail to maintain the DNA damage-induced arrest, and gradually escape the checkpoint and progress into the M phase, when they eventually undergo aberrant mitoses (Bunz *et al*, 1998). For this p53-mediated checkpoint arrest, p53 may exert its functions via transactivation of its target genes, such as *p21/Cip1*, *GADD45*, *14-3-3σ*, or via transrepression of *cdc2* and *cyclin B1* genes (el-Deiry *et al*, 1994; Hermeking *et al*, 1997; Bunz *et al*, 1998; Taylor *et al*, 1999; Wang *et al*, 1999). Therefore, once activated by the DNA damage signal, p53 blocks Cdc2-cyclin B kinase activity to prevent completely the entry of damaged chromosomes into mitosis.

In a previous study with normal human mammary epithelial cells (HMECs), we have shown that overexpression of the protooncogene c-*myc* significantly attenuates the DNA damage-induced G1/S arrest, suggesting that deregulated c-Myc predisposes normal cells to genomic instability (Sheen and Dickson, 2002). In addition, we also identified the formation of micronuclei, selectively in cells with excess c-Myc activity, following a prolonged interval, post-treatment with ionising radiation (IR). These micronuclei result from aberrant mitoses, following various types of deleterious stresses to cells (Forrester *et al*, 1999,^2000^). Depending on the cell line and the nature of the damage, the resulting mitoses are aberrant and incomplete, skipping a proper segregation of chromosomes and cytokinesis. These abortive mitoses eventually produce multiple micronuclei, which are completely, or partially, separated from each other (Forrester *et al*, 1999,^2000^). Although their underlying mechanisms are not fully understood, the aberrant mitoses can be induced not only by DNA damage but also by inappropriate activation of Cdc2-cyclin B mitotic kinase (Heald *et al*, 1993), suggesting that micronuclei are resulting from an unprepared or inappropriate entry into mitosis. These aberrant mitoses also suggest that an abrogation of G2/M-entry checkpoint precedes the micronucleation. Based on these ideas, we hypothesised that the overexpression of c-Myc may alter the G2/M arrest, following DNA damage, leading to an inappropriate entry of damaged chromosomes into mitosis.

## RESULTS

### Overexpression of c-Myc has no effect on the overall time-course of the nocodazole-induced mitotic arrest

To measure cellular entry into mitosis from the G2 phase, we employed the mitotic-arrest assay (Bunz *et al*, 1998). Cells arrest in mitosis, following treatment with a microtubule (MT) destabiliser (e.g. colchicine or nocodazole) because the drug destroys an MT-based mitotic spindle that is essential for mitosis. When DNA damage is introduced, normal cells, however, do not arrest in mitosis, even in the presence of MT destabiliser. Instead, they arrest at G2/M because of the DNA damage-induced checkpoint. However, cells defective in their G2/M checkpoint should produce mitotic cells, following DNA damage, which undergo inappropriate entry into mitosis. Therefore, one can test the intactness of DNA damage-induced G2/M checkpoint by measuring mitotic figures, following the introduction of DNA damage, in the presence of MT destabiliser. One condition of this mitotic-arrest assay would be that experimental populations should arrest in mitosis over the same time-course following the MT destabiliser treatment. In other words, all samples should possess a similarly effective spindle-assembly checkpoint that monitors the intactness of mitotic spindle assembly and governs mitotic progress.

**Figure 1:**
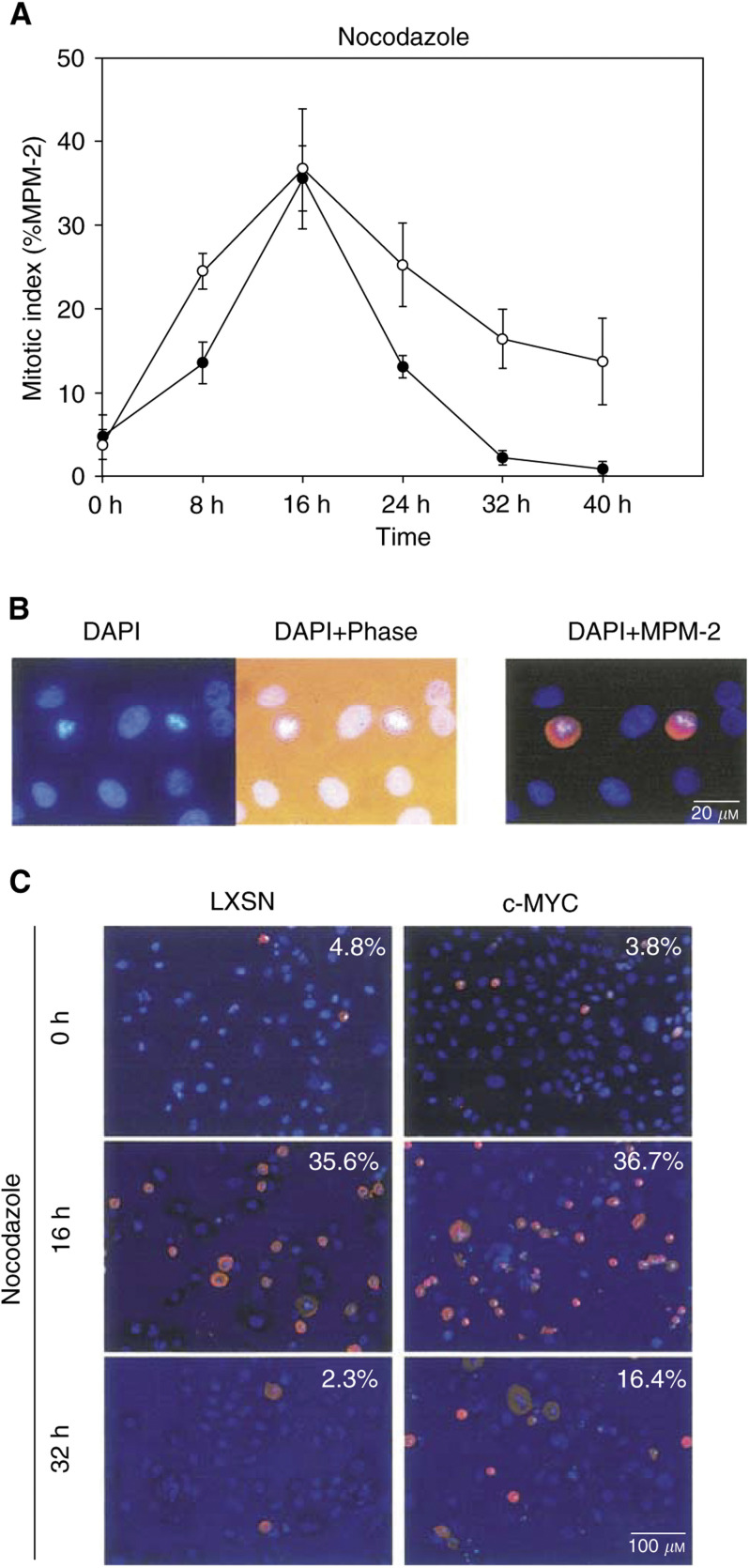
c-Myc has no effect on the time-course of nocodazole-induced mitotic arrest of normal HMECs. Following an infection with LXSN vector-only or c-Myc retroviruses, cells were allowed to express exogenous genes for 48 h. (**A**) Cells were treated with nocodazole, an MT destabiliser, and their degree of arrest in mitosis (%MPM-2 positive) was measured over a time-course of 0, 8, 16, 24, 32, and 40 h time-points. Both LXSN-infected controls (filled circle) and c-Myc-overexpressing cells (nonfilled circle) arrested at mitosis over the same time-course, following artificial abrogation of mitotic spindles. (**B**) DAPI staining identifies mitotic cells, with typical, condensed chromosomes. Rounded-up cells, on phase-contrast microscopy, match with cells having condensed chromosomes on DAPI staining. Finally, mitotic cells were confirmed with a mitosis-specific marker, MPM-2. (**C**) Representative figures demonstrate gradual changes of MPM-2-positive mitotic cells, following prolonged treatment with nocodazole.

We initially tested the effects of c-Myc on the MT destabiliser-induced spindle assembly checkpoint, by measuring mitotic figures with the MPM-2 marker, following treatment of nocodazole. MPM-2, a mitosis-specific marker (Davis *et al*, 1983), has been used to identify mitotic cells with rounded-up morphology and condensed chromosomes, representative characteristics of mitosis (Figure 1B

). In independently repeated experiments, both retroviral vector (LXSN)-infected control cells and c-Myc-infected cells displayed mitotic arrest over the same time-course, following nocodazole treatment (Figure 1A and C). After prolonged arrest in mitosis, cells appeared to adapt to the absence of a mitotic spindle and escaped from mitosis. This is consistent with the previously reported ‘mitotic slippage’ phenomena following cellular treatment with mitotic spindle inhibitors (Schimke *et al*, 1991,^1994^).

### Overexpression of c-Myc attenuates the DNA damage-induced G2/M-entry checkpoint

**Figure 2:**
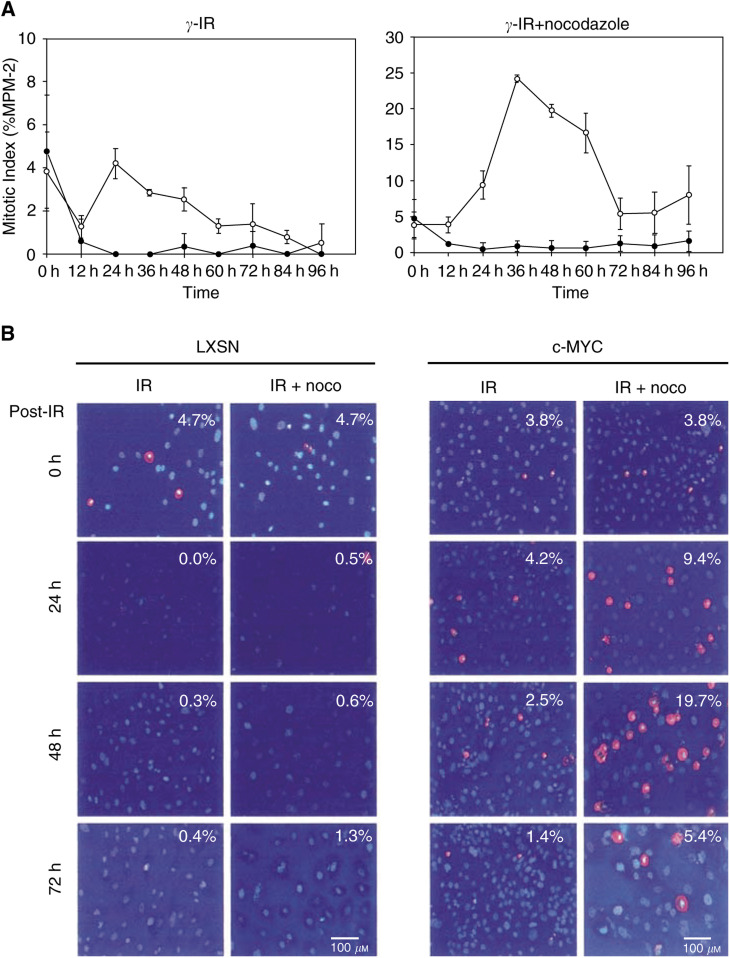
Overexpression of c-Myc attenuates DNA damage-induced G2/M arrest. To investigate the G2/M transition, after DNA damage, cells were irradiated with 10 Gy, and MPM2-positive cells were measured over a time-course. (**A**) Irradiated LXSN-infected controls (filled circle) and c-Myc-overexpressing cells (nonfilled circle) were stained with MPM2 in the absence or presence of nocodazole. Note the different scale of the *y*-axis (mitotic index) in the two graphs. (**B**) Representative figures, showing a gradual increase of MPM2-positive ones, selectively in irradiated c-Myc-overexpressing cells.

In asynchronous populations, with a certain percentage (∼15–20%) of cells in G2, both LXSN-infected control cells and Myc-infected cells displayed a positive index of MPM-2, indicating mitosis at a frequency of 4–5% (Figure 2A and B

). The introduction of DNA damage (10 Gy of *γ*-irradiation) led LXSN-infected controls to decrease their mitotic index dramatically, and led irradiated cells to display almost no mitotic cells. In contrast, c-Myc-infected cells regained a mitotic index (∼4%) that is comparable to the untreated population at the 24 h time-point, following an initial decrease at the 12 h time-point. Following 24 h, c-Myc-overexpressing cells progressively decreased their mitotic index. This attenuated DNA damage-induced G2/M arrest, in c-Myc-overexpressing cells, was even more evident, following the introduction of DNA damage in the presence of the MT destabiliser (nocodazole) (Figure 2A and B). LXSN-infected controls consistently displayed a decreased mitotic index, following *γ*-irradiation, regardless of nocodazole treatment. However, in the presence of nocodazole-mediated mitotic arrest, c-Myc-infected cells showed an increasing frequency of MPM-2-positive mitotic figures, following *γ*-irradiation, and reached a peak at 36 h time-point (Figure 2A).

### Cells with deregulated c-Myc display high levels of cyclin B1 before and after *γ*-irradiation

**Figure 3:**
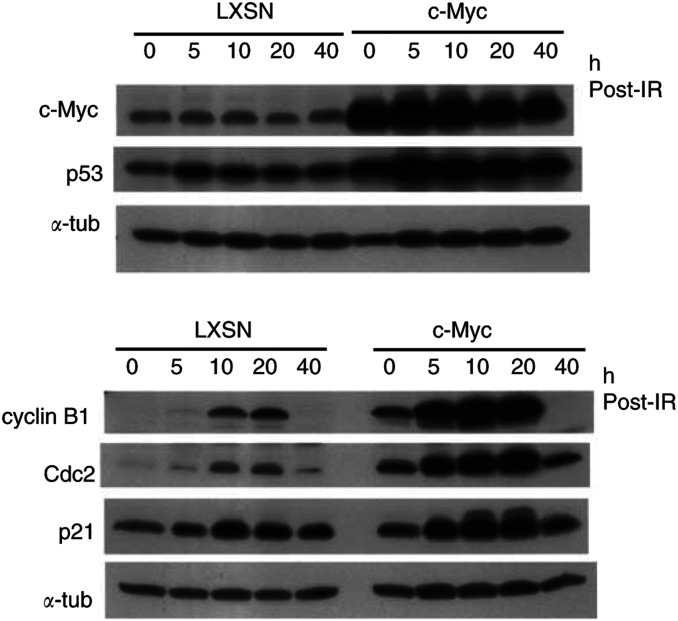
Overexpression of c-Myc upregulates cyclin B1 and results in an inappropriately high level of cyclin B1, following DNA damage. Western blotting analysis of c-Myc, p53, cyclin B1, Cdc2, and p21/Cip1 in normal HMECs over a time-course, following 10 Gy *γ*-irradiation. *α*-tubulin blots serve the equal loading control.

To gain insights into the molecular mechanism(s) for the c-Myc-mediated attenuation of the G2/M checkpoint, we measured changes in the levels of proteins that are implicated in the G2/M transition, over a time-course, following IR (Figure 3

). After transient expression of c-Myc in normal HMECs for 48 h, cells were immediately irradiated with 10 Gy IR, and cell lysates were prepared. The ectopic expression of c-Myc led cells to express higher levels of p53 and p21 proteins, even in the absence of DNA damage, and made them more responsive to the DNA damage. On the other hand, consistent with previous studies in our group (Sheen and Dickson, 2002), transient expression of c-Myc did not inhibit or arrest the cell cycle progression of normal HMECs. It consistently led to a cell cycle profile characterised by a lower percentage of G1 and a higher percentage of S, when compared to that of LXSN-infected controls. Significantly, we identified surprisingly high levels of cyclin B1 protein in normal HMECs overexpressing c-Myc, and these upregulated levels were maintained for a period of time, even after DNA damage (Figure 3). As we also identified high levels of the Cdc2 kinase itself in c-Myc-overexpressing cells, we wanted to determine whether c-Myc controls expression of these critical G2/M genes at the mRNA level or at a post-mRNA level. In assays employing a cDNA microarray, we were able to detect a markedly high level of cyclin B1 (CCNB1) gene transcripts (Table 1

**Table 1:** cDNA array analysis of changes in gene expression, following IR

**Gene name**	**LXSN-0 h**	**LXSN-10 h**	**LXSN-20 h**	**MYC-0 h**	**MYC-10 h**	**MYC-20 h**
Bax	1.00	1.60	1.56	2.85	2.18	2.76
Cyclin B	1.00	1.49	0.98	3.60	2.32	2.71
Cyclin D1	1.00	1.84	1.80	1.14	0.79	0.91
Cyclin D2	1.00	0.95	1.22	1.33	1.19	1.44
Cdk1 (cdc2)	1.00	1.15	0.67	1.54	1.59	1.63
Cdk2	1.00	0.56	0.49	1.52	1.40	2.44
Cdk4	1.00	0.73	0.60	0.97	1.20	1.22
p53	1.00	0.47	1.79	0.45	1.63	0.73
p21/Cip1	1.00	1.82	1.39	1.57	1.99	2.05
p16ink4	1.00	0.63	0.55	1.07	0.77	0.82
p19Ink4d	1.00	1.35	0.97	0.72	1.15	0.81
GADD45	1.00	1.34	1.27	1.35	0.98	1.89
Mdm2	1.00	1.71	1.77	2.01	2.86	4.54

Human cell-cycle GEArray™ Q series kit was used to identify changes in mRNA levels following *γ*-irradiation. Fold differences in mRNA levels to the control group (HMEC-LXSN) were calculated. The GEArray data were quantitated with the ImageQuaNT™ software and analysed with the GEArray analyzer™ 1.2 software. Background signals were processed with minimal value and positive signals were normalised with the median value.

IR=ionising radiation.

). We also detected a marginally higher level of Cdc2 transcripts.

### Overexpression of c-Myc sensitises normal HMECs to the DNA damage-induced apoptosis

**Figure 4:**
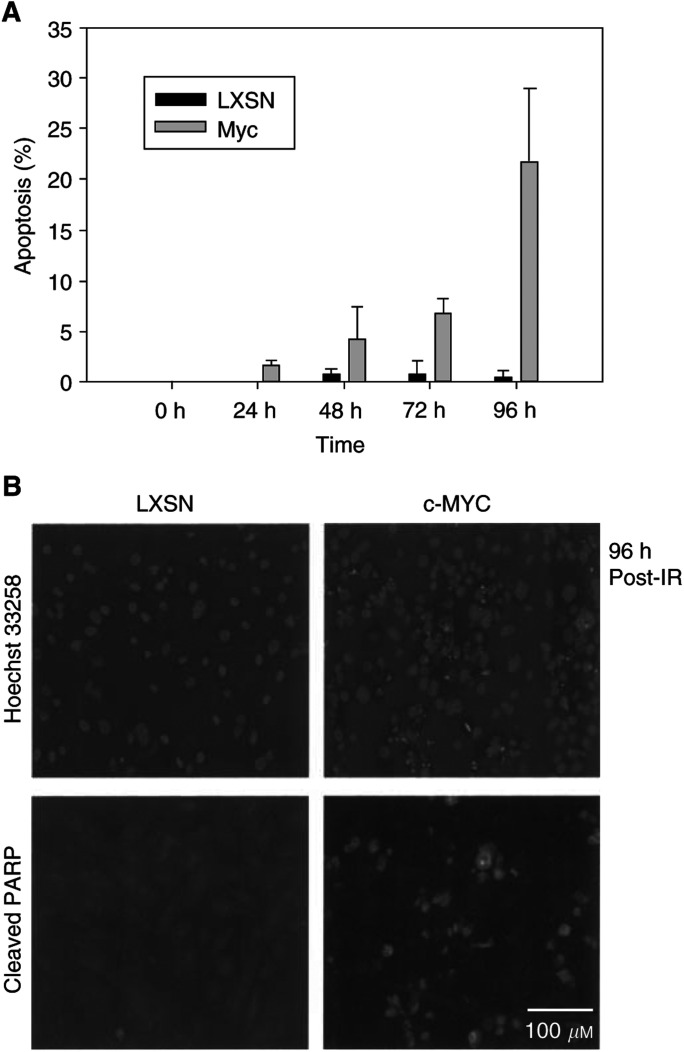
c-Myc-overexpressing cells are sensitive to DNA damage-induced apoptosis. DNA damage eventually induces apoptosis of c-Myc-overexpressing cells. (**A**) A time-course change in the percentage of apoptotic cells was measured with Hoechst 33258 and cleaved PARP staining. (**B**) Representative figures, showing apoptotic cells with fragmented DNA, in Hoechst 33258 staining and with cleaved PARP.

In our experiments using a mitotic-arrest assay to study the G2/M transition, we frequently identified condensed nuclei, which are stained with DAPI, but not with MPM-2, following DNA damage (Figure 2B). Therefore, we wanted to determine if these condensed nuclei indicate apoptotic cells, by staining with an antibody against cleaved PARP. Full-length PARP proteins are cleaved during the apoptosis process. Following a prolonged incubation after IR-induced DNA damage, putative apoptotic nuclei, with fragmented chromosomes, were identified selectively in c-Myc-overexpressing cells; the apoptotic phenotype of these cells was confirmed with staining for cleaved PARP (Figure 4

). Based on these results, we concluded that c-Myc-overexpressing cells are selectively sensitive to the DNA damage-induced apoptosis.

## DISCUSSION

According to a recent study of *c-myc* (−/−) rat fibroblasts, the absence of c-Myc prolongs both G1 and G2, suggesting a role for c-Myc in the G2/M transition (Mateyak *et al*, 1999). Furthermore, we previously showed that some cells, with deregulated c-Myc activity, replicated their damaged templates and ended up in post-M phase with multiple micronuclei, which suggests the possibility that deregulated c-Myc attenuates the DNA damage-induced G2/M checkpoint (Sheen and Dickson, 2002).

We employed the mitotic-arrest assay to measure G2/M entry, following DNA damage (Bunz *et al*, 1998). One critical condition for the mitotic-arrest assay is that all sample groups should have the same kinetics of mitotic arrest and escape from the arrest, following nocodazole treatment, because the nocodazole-induced chemical blockage is not complete. Cells eventually adapt to this nocodazole challenge and escape to a state, termed ‘G1-like post-M phase’, with interphase morphology with micronuclei (‘mitotic slippage’ phenotype) (Schimke *et al*, 1991,^1994^). Therefore, we first demonstrated that both control LXSN- and c-Myc-infected normal HMECs have the same kinetics of mitotic arrest and escape, following nocodazole treatment (Figure 1). At the same time, our results also provided a clue on how MT destabiliser induces polyploidy in c-Myc-overexpressing cells. As c-Myc-overexpressing cells did not escape, any faster than control cells from the nocodazole-induced mitotic-arrest, deregulated c-Myc has no effects on the spindle-assembly checkpoint, suggesting the possibility of c-Myc's effects selectively on the post-M checkpoint. Similarly, p53-deficient cells have a normal spindle-assembly checkpoint and an altered post-M checkpoint (Lanni and Jacks, 1998; Li and Dang, 1999).

We irradiated unsynchronised populations in order to avoid any effects of artificial synchronisation on the cell cycle of normal HMECs. Following IR, the empty vector control cells (HMEC-LXSN) displayed a dramatic reduction of mitoses, regardless of nocodazole treatment. However, in surprising contrast, c-Myc-overexpressing cells showed a significant number of mitotic cells after *γ*-irradiation. The effects were even clearer in the presence of nocodazole (Figure 2A and B). We interpreted these results to indicate that c-Myc attenuates G2/M arrest, following DNA damage, and that the cells undergoing inappropriate mitoses were arrested in M phase.

In Western analyses, we demonstrated that cyclin B1 and Cdc2, two components of the cyclin B1/Cdc2 mitotic kinase complex, are significantly upregulated, following a transient overexpression of c-Myc in normal HMECs. The upregulated levels of cyclin B1 and Cdc2 were maintained, even after DNA damage, until they eventually began to decrease (Figure 3). Next, the markedly high level of cyclin B1 mRNA transcripts was identified in a cDNA array analysis (Table 1). A previous study reported the effects of overexpression of cyclin B1 mutants on the DNA damage-induced G2/M checkpoint in HeLa cells (Jin *et al*, 1998). In this report, the overexpression of wild-type cyclin B1 was initially considered insufficient to attenuate G2/M arrest, following DNA damage. However, in the presence of nocodazole, its effects were significantly demonstrated (Jin *et al*, 1998). In the current study, we showed a significantly attenuated G2/M arrest in c-Myc-overexpressing HMECs that coincidently have upregulated levels of cyclin B1.

The overexpression of c-Myc alone was not sufficient to induce premature mitosis; this may be primarily because the inhibitory phosphorylations of Cdc2 are independently regulated (Jin *et al*, 1996). However, we cannot rule out a possible role of the c-Myc-mediated alteration of G2/M arrest in c-Myc-induced genomic instability. The high-fidelity performance of mitosis is extremely critical for an accurate segregation of genetic materials and for the maintenance of genomic stability. Presumably, the unprepared entry into mitosis, such as what may occur during premature mitosis, may produce a significant amount of genomic damage. However, severe genomic damage would be fatal and cells sustaining such damage from premature mitosis may not survive. On the other hand, attenuated control over the G2/M arrest, which is still unsatisfactory for the precise segregation of genes, could be barely tolerable for cell survival, such that cells containing genomic alterations could survive.

In addition to observing a checkpoint alteration, we found that normal HMECs with c-Myc overexpression eventually undergo apoptosis, following DNA damage, indicating that deregulated c-Myc sensitises cells to apoptosis (Figure 4). The parental, normal HMECs were resistant to the DNA damage-induced apoptosis, presumably because of the arrest of their cell cycle. Although c-Myc has been known to induce apoptosis under cellular stresses, such as growth factor deprivation, we currently do not fully understand as to how c-Myc sensitises cells or what triggers apoptosis in c-Myc-overexpressing cells, after DNA damage.

As shown in Figure 3, c-Myc slightly upregulates p53, and c-Myc-overexpressing cells rapidly respond to DNA damage by inducing p21/Cip1, suggesting the possibility of heightened p53 activity and p53-mediated apoptosis of c-Myc-overexpressing cells. This hypothesis would be compatible with a c-Myc-mediated checkpoint attenuation phenotype, if c-Myc modulates or redirects p53 toward apoptosis, rather than toward cell cycle arrest. An alternative hypothesis would be that abrogation of cell cycle arrest precedes apoptosis. In p21(−/−) human colon carcinoma cells, the elimination of p21 makes the cells dramatically susceptible to DNA damage-induced apoptosis, compared to the parental cells (Waldman *et al*, 1996). In a pilot experiment, however, we failed to rescue c-Myc-overexpressing cells from apoptosis, following DNA damage, with an induced cell cycle arrest. Instead, we observed that induction of artificial arrest of cell cycle, with excess thymidine or Purvalanol, led to apoptosis of c-Myc-overexpressing HMECs (data not shown). The prior reports showed that c-Myc-overexpressing fibroblasts are highly apoptotic when cell cycles are artificially perturbed (Evan *et al*, 1992). Therefore, future studies, focusing on molecular mechanisms for c-Myc-induced checkpoint alteration and sensitisation to apoptosis, are required to determine how the two remarkable Myc phenotypes are related in terms of different c-Myc target genes.

## MATERIALS AND METHODS

### Cell culture and retroviral infection

Normal, finite lifespan HMECs (#1001-12, CC-2551) at passage 7, originally derived from reduction mammoplasty tissues, were obtained from BioWhittaker, Inc. (Walkersville, MD, USA), and cells between passage 8 and 10 were used for experiments. Normal HMECs were maintained according to the supplier's instructions in mammary epithelial cell growth medium (MEGM, CC-3152), supplemented with 52 *μ*g ml^−1^ bovine pituitary extract, 10 ng ml^−1^ human EGF, 5 *μ*g ml^−1^ insulin, and 0.5 *μ*g ml^−1^ hydrocortisone, and were grown in 37°C incubators with low (0.1–0.2%) CO_2_ settings. Transient overexpression of c-*myc* was performed with pLXSN-Myc as previously described (Sheen and Dickson, 2002). Briefly, cells were plated 1 day before retroviral infection at ∼70% confluency. Transient infection was conducted with recombinant retroviruses, at an MOI of 3–5. Following 12 h of infection, in the presence of 8 *μ*g ml^−1^ polybrene, and 48 h of gene expression, infected cells were utilised for experiments, without further selection in G418. For the mitotic-arrest assay, culture media were replaced, following *γ*-irradiation, with fresh media, containing nocodazole (0.15 *μ*g ml^−1^), and cells were further incubated.

### Induction of DNA damage

DNA damage was induced by IR, using a ^137^Cs source *γ*-irradiator (JL Shepherd Mark-I Irradiator, San Fernando, CA, USA), at a rate of 3.6 Gy min^−1^, until the specified, absorbed dosage was reached. For irradiation of attached cells, cells were plated in culture dishes on Day 1, and irradiated at 70–80% confluency on Day 2. All experimental samples in dishes were kept rotating on a turntable, inside the irradiator machine, during the irradiation procedure.

### Immunocytochemistry

Mitotic cells were monitored by staining with MPM-2, a mitosis-specific marker. After *γ*-irradiation, cells were plated on glass cover slips in prewarmed media in 12-well culture plates. A total of 5 × 10^4^ cells were plated per well and then allowed to grow in the presence or absence of nocodazole. At each time-point, cells were washed three times with PBS (10 min each wash) and fixed in 4% paraformaldehyde/PBS for 20 min. Next, the fixed cells were permeabilised with 0.5% Triton X-100/PBS. Following incubation with an anti-MPM-2 monoclonal antibody (10 *μ*g ml^−1^, Upstate Biotech, Waltham, MA, USA), cells were washed three times with PBS and the nuclei were counterstained with 4′,6′-diamidino-2-phenylindole (DAPI), for 5 min. Finally, after several additional PBS washes, the cover clips were mounted on glass slides, with Prolong mounting medium (Molecular Probes, Eugene, OR, USA) for fluorescence microscopy. Quantitative analysis of immunofluorescence data was carried out with Image-Pro® Plus software (Media Cybernetics, Silver Spring, MD, USA).

### Western blot analysis

Cell lysates were prepared in 2 × Laemmli sample buffer, containing 1% SDS and *β*-mercaptoethanol. After collecting approximately 10^6^ cells in 100 *μ*l of sample buffer, whole cell lysates were boiled for 10 min, cleared by centrifugation at 4°C, and stored at −80°C until use. For SDS–polyacrylamide gel electrophoresis, 20 *μ*l of the cleared lysates was loaded into a well of the gel. Separated proteins were transferred onto an Immobilon-P polyvinylidene fluoride membrane (Millipore, Bedford, MA, USA). After tank transfer, membranes were blocked for 1 h at room temperature in 0.05% Tween 20-PBS (PBS-T) containing 5%, nonfat dried milk, and then probed with primary antibody diluted in PBS-T overnight in a 4°C cold room. Antibodies and concentrations were as follows: human c-Myc (9E10, 2 *μ*g ml^−1^; 14851A Pharmingen, San Diego, CA, USA), human p53 (PAb1801, 2.5 *μ*g ml^−1^; Ab-2 Oncogene Research Products, Boston, MA, USA), p21/WAF1 (EA10, 1 *μ*g ml^−1^; Ab-1 Oncogene Research Products), cyclin B1 (clone GNS1, 1 *μ*g ml^−1^; Ab-3 NeoMarkers, Fremont, CA, USA), Cdc2/cdk1 (A17.1.1+POH-1, 1 *μ*g ml^−1^; Ab-3 NeoMarkers), and *α*-tubulin (DM1A, 1 : 1000 dilution; Ab-2 NeoMarkers). The blots were then washed with PBS-T three times for 10 min. All blots were next incubated in Horse Radish Peroxidase-conjugated secondary goat anti-mouse or anti-rabbit antibody (BioRad, 1 : 3000 dilution) for 1 h at room temperature for chemiluminescent detection.

### cDNA microarray analysis

For measuring changes in mRNA levels, following *γ*-irradiation, we used the Human Cell-cycle GEArray™ Q series kit (HS-001; SuperArray Inc., Bethesda, MD, USA). Following the preparation of total RNA with the Trizol® reagent (Invitrogen™/Life Technologies, Carlsbad, CA, USA), cDNA probes were synthesised and hybridised to the GEArray membranes according to the provider's instructions. The hybridisation signals were digitised with Phosphorimager™ 445 SI (Molecular Dynamics, Sunnyvale, CA, USA) and data were quantitated and analysed with ImageQuaNT™ software (Molecular Dynamics, Sunnyvale, CA, USA) and the GEArray analyzer™ 1.2 software (SuperArray Inc., Bethesda, MD, USA).

## References

[bib1] Bunz F, Dutriaux A, Lengauer C, Waldman T, Zhou S, Brown JP, Sedivy JM, Kinzler KW, Vogelstein B (1998) Requirement for p53 and p21 to sustain G2 arrest after DNA damage. Science 282: 1497–1501 9822382 10.1126/science.282.5393.1497

[bib2] Davis FM, Tsao TY, Fowler SK, Rao PN (1983) Monoclonal antibodies to mitotic cells. Proc Natl Acad Sci USA 80: 2926–2930 6574461 10.1073/pnas.80.10.2926 PMC393946

[bib3] El-Deiry WS, Harper JW, O'Connor PM, Velculescu VE, Canman CE, Jackman J, Pietenpol JA, Burrell M, Hill DE, Wang Y, Wiman KG, Mercer WE, Kastan MB, Kohn KW, Elledge SJ, Kinzler KW, Vogelstein B (1994) WAF1/CIP1 is induced in p53-mediated G1 arrest and apoptosis. Cancer Res 54: 1169–1174 8118801

[bib4] Evan GI, Wyllie AH, Gilbert CS, Littlewood TD, Land H, Brooks M, Waters CM, Penn LZ, Hancock DC (1992) Induction of apoptosis in fibroblasts by c-myc protein. Cell 69: 119–128 1555236 10.1016/0092-8674(92)90123-t

[bib5] Forrester HB, Albright N, Ling CC, Dewey WC (2000) Computerized video time-lapse analysis of apoptosis of REC:Myc cells X-irradiated in different phases of the cell cycle. Radiat Res 154: 625–639 11096419 10.1667/0033-7587(2000)154[0625:cvtlao]2.0.co;2

[bib6] Forrester HB, Vidair CA, Albright N, Ling CC, Dewey WC (1999) Using computerized video time lapse for quantifying cell death of X-irradiated rat embryo cells transfected with c-myc or c-Ha-ras. Cancer Res 59: 931–939 10029087

[bib7] Graves PR, Lovly CM, Uy GL, Piwnica-Worms H (2001) Requirement for p53 and p21 to sustain G2 arrest after DNA damage. Oncogene 20: 1839–1851 11313932 10.1038/sj.onc.1204259

[bib8] Hartwell L (1992) Defects in a cell cycle checkpoint may be responsible for the genomic instability of cancer cells. Cell 71: 543–546 1423612 10.1016/0092-8674(92)90586-2

[bib9] Hartwell L, Weinert T, Kadyk L, Garvik B (1994) Cell cycle checkpoints, genomic integrity, and cancer. Cold Spring Harb Symp Quant Biol 59: 259–263 7587077 10.1101/sqb.1994.059.01.030

[bib10] Heald R, McLoughlin M, McKeon F (1993) Human wee1 maintains mitotic timing by protecting the nucleus from cytoplasmically activated Cdc2 kinase. Cell 74: 463–474 8348613 10.1016/0092-8674(93)80048-j

[bib11] Hermeking H, Lengauer C, Polyak K, He TC, Zhang L, Thiagalingam S, Kinzler KW, Vogelstein B (1997) 14-3-3 sigma is a p53-regulated inhibitor of G2/M progression. Mol Cell 1: 3–11 9659898 10.1016/s1097-2765(00)80002-7

[bib12] Jin P, Gu Y, Morgan DO (1996) Role of inhibitory CDC2 phosphorylation in radiation-induced G2 arrest in human cells. J Cell Biol 134: 963–970 8769420 10.1083/jcb.134.4.963 PMC2120957

[bib13] Jin P, Hardy S, Morgan DO (1998) Nuclear localization of cyclin B1 controls mitotic entry after DNA damage. J Cell Biol 141: 875–885 9585407 10.1083/jcb.141.4.875 PMC2132764

[bib14] Kastan MB (2001) Cell cycle. Checking two steps. Nature 410: 766–767 11298430 10.1038/35071218

[bib15] Lanni JS, Jacks T (1998) Characterization of the p53-dependent postmitotic checkpoint following spindle disruption. Mol Cell Biol 18: 1055–1064 9448003 10.1128/mcb.18.2.1055 PMC108818

[bib16] Li Q, Dang CV (1999) c-Myc overexpression uncouples DNA replication from mitosis. Mol Cell Biol 19: 5339–5351 10409725 10.1128/mcb.19.8.5339 PMC84377

[bib17] Mateyak MK, Obaya AJ, Sedivy JM (1999) c-Myc regulates cyclin D-Cdk4 and -Cdk6 activity but affects cell cycle progression at multiple independent points. Mol Cell Biol 19: 4672–4683 10373516 10.1128/mcb.19.7.4672 PMC84265

[bib18] Murray A, Hunt T (1993) The Cell Cycle: An Introduction. Oxford: Oxford University Press

[bib19] Paulovich AG, Toczyski DP, Hartwell LH (1997) When checkpoints fail. Cell 88: 315–321 9039258 10.1016/s0092-8674(00)81870-x

[bib20] Peng CY, Graves PR, Thoma RS, Wu Z, Shaw AS, Piwnica-Worms H (1997) Mitotic and G2 checkpoint control: regulation of 14-3-3 protein binding by phosphorylation of Cdc25C on serine-216. Science 277: 1501–1505 9278512 10.1126/science.277.5331.1501

[bib21] Sanchez Y, Wong C, Thoma RS, Richman R, Wu Z, Piwnica-Worms H, Elledge SJ (1997) Conservation of the Chk1 checkpoint pathway in mammals: linkage of DNA damage to Cdk regulation through Cdc25. Science 277: 1497–1501 9278511 10.1126/science.277.5331.1497

[bib22] Schimke RT, Kung A, Sherwood SS, Sheridan J, Sharma R (1994) Life, death and genomic change in perturbed cell cycles. Philos Trans R Soc Lond B Biol Sci 345: 311–317 7846128 10.1098/rstb.1994.0111

[bib23] Schimke RT, Kung AL, Rush DF, Sherwood SW (1991) Differences in mitotic control among mammalian cells. Cold Spring Harb Symp Quant Biol 56: 417–425 1819503 10.1101/sqb.1991.056.01.049

[bib24] Sheen JH, Dickson RB (2002) Overexpression of c-Myc alters G(1)/S arrest following ionizing radiation. Mol Cell Biol 22: 1819–1833 11865060 10.1128/MCB.22.6.1819-1833.2002 PMC135614

[bib25] Taylor WR, DePrimo SE, Agarwal A, Agarwal ML, Schonthal AH, Katula KS, Stark GR (1999) Mechanisms of G2 arrest in response to overexpression of p53. Mol Biol Cell 10: 3607–3622 10564259 10.1091/mbc.10.11.3607 PMC25646

[bib26] Taylor WR, Stark GR (2001) Regulation of the G2/M transition by p53. Oncogene 20: 1803–1815 11313928 10.1038/sj.onc.1204252

[bib27] Waldman T, Lengauer C, Kinzler KW, Vogelstein B (1996) Uncoupling of S phase and mitosis induced by anticancer agents in cells lacking p21. Nature 381: 713–716 8649519 10.1038/381713a0

[bib28] Wang XW, Zhan Q, Coursen JD, Khan MA, Kontny HU, Yu L, Hollander MC, O'Connor PM, Fornace AJJ, Harris CC (1999) GADD45 induction of a G2/M cell cycle checkpoint. Proc Natl Acad Sci USA 96: 3706–3711 10097101 10.1073/pnas.96.7.3706 PMC22358

[bib29] Zeng Y, Piwnica-Worms H (1999) DNA damage and replication checkpoints in fission yeast require nuclear exclusion of the Cdc25 phosphatase via 14-3-3 binding. Mol Cell Biol 19: 7410–7419 10523629 10.1128/mcb.19.11.7410 PMC84734

[bib30] Zhao H, Piwnica-Worms H (2001) ATR-mediated checkpoint pathways regulate phosphorylation and activation of human Chk1. Mol Cell Biol 21: 4129–4139 11390642 10.1128/MCB.21.13.4129-4139.2001 PMC87074

